# Major contributors to FLASH sparing efficacy emerge from murine skin studies: dose rate, total dose per fraction, anesthesia and oxygenation

**DOI:** 10.3389/fonc.2024.1414584

**Published:** 2024-10-25

**Authors:** Brian W. Pogue, William S. Thomas, Armin D. Tavakkoli, Lesley A. Jarvis, P. Jack Hoopes

**Affiliations:** ^1^ Department of Medical Physics, University of Wisconsin-Madison, Madison, WI, United States; ^2^ Thayer School of Engineering at Dartmouth, Hanover, NH, United States; ^3^ Department of Surgery, Geisel School of Medicine at Dartmouth, Hanover, NH, United States

**Keywords:** radiotherapy, skin, FLASH radiotherapy, dosimetry, radiobiology, radiation response biomarkers, radiation response

## Abstract

**Background:**

Normal tissue sparing from radiation damage upon ultra-high dose rate irradiation, known as the FLASH effect with an equivalent tumor response, has been widely reported in murine skin models, and translation of this type of radiotherapy to humans has already begun, with skin sparing being a primary outcome expected.

**Methods:**

This study reviews the status of the field, focusing on the proposed mechanisms and skin response assays, outlining what has become known in terms of input parameters that might control the magnitude of the FLASH effect.

**Results:**

Murine studies have largely focused on acute damage responses, developing over 3–8 weeks, to single doses of FLASH versus conventional dose rate (CDR), suggesting that at dose rates above tens of Gray per second, with a total dose of more than 20 Gy, the FLASH effect is induced. Fractionated delivery appears to be possible, although fraction sizes >17 Gy appear to be needed for sparing efficacy. The interplay between the dose rate and total dose per fraction remains to be fully elucidated. Oxygen is a modulator of efficacy, with both hypoxia and hyperoxia diminishing the effect of FLASH. Measurement of transient changes in oxygen levels is possible and may be a marker of treatment efficacy.

**Conclusion:**

Taken together, murine skin data provide important information for translational studies, despite the associated limitations. Studies of later-term sparing effects, as well as studies on pig skin, are needed to take the next step in assessing translational FLASH efficacy. The control of biological factors, such as tissue oxygenation, may be required to understand and control the response.

## 1 Introduction

Discovery of the ‘FLASH effect,’ which comprises normal tissue sparing in radiation therapy (RT) with an equivalent tumor response when delivered at ultra-high dose rate (UHDR) (1–3), termed FLASH RT, has spurred significant interest in testing this phenomenon in translational studies (4–6). Larger cohort human trials have already been started for skin cancers with electron UHDR RT (7) and bone metastases with proton UHDR RT (8). Other phase I trials began at multiple research centers. While the initial focus of many early phase trials will be to assess the safety of FLASH RT, the most interesting efficacy measure will be the extent of normal skin sparing (9). The utility of the skin as a normal tissue to determine sparing is due to two main reasons: 1) the skin is a low-risk and relatively easy to assay organ, and 2) nearly all radiation treatments, without intraoperative delivery, necessarily involve normal skin. Skin irradiation is also a good match for electron FLASH studies, where the penetration of electron depth is modest, or for shoot-through proton irradiation, where there is no benefit from a Bragg peak in the tumor, leading to higher skin doses (10–12). Skin damage as a measure is inherently safer for patients than damage to most other organ systems, which could potentially reveal the sparing effect (13, 14), such as the lung, brain, or colon (1–3, 15). Skin damage is also relatively easy to assay using numerous non-invasive tools, such as dermatoscopes and subclinical erythema meters, which are readily available in dermatology clinics. Skin erythema is a well-characterized biological function, both in timeline and severity, characterized by reddening of the skin to its extreme, moist desquamation (16). Pre-clinically, most skin irradiation studies focus on moist desquamation or ulceration measured in % of subjects or at a defined time point as quantitative endpoints. Given that nearly all systematic skin response data were obtained using murine models, the murine skin response to RT was reviewed here with the goal of quantifying and establishing the key factors that affect the efficacy of FLASH sparing.

The dominant factors that appear to affect the magnitude of the FLASH effect are still debated, but there appears to be agreement that the dose rate (17, 18) and the total dose delivered per treatment are among the key parameters (19). These two factors are not necessarily linked, although it might be surprising if they were not, but they appear to be implicated in the magnitude of skin-sparing efficacy. The fractionation relationship with the total dose per treatment is equally important because of fractionation relationships with late effects (20, 21) and discrepancies between the onset of acute effects when acute time points such as erythema tend to be dose-independent. Another key factor that has emerged in the last couple of years is the level of oxygenation (22–24), which is quantified by the inspired gas level, choice of anesthesia, or direct measurement of tissue oxygen. Tissue oxygenation is likely to be the key contributing factor; however, inspired gas is often used as the observed factor for simplicity. Tissue oxygen is complex to measure, but it may be critical because it can be highly variable and uncontrolled in murine skin studies. However, methods to quantify tissue oxygenation have emerged such that it is possible to measure it *in vivo*, and in fact, oxygen is one of the few biological factors that can be reliably measured *in situ* at the time of irradiation (25–27). Data on the type of anesthesia, carrier gas, and direct oxygenation measurement were examined in published murine FLASH studies.

The hypothesis here is that most causal conclusions about skin FLASH effects must be inferred from murine studies because relatively little has been published on the FLASH effect in skin in species higher than mice with systematic variation of these input parameters. There are single-arm studies in veterinary medicine and limited porcine data (28), but little mechanistic information can lead to conclusions about dose rate, total dose, or oxygenation parameters, and how they might affect isodose skin-sparing trials. The goal of this study was to outline as many definitions of the input control parameters as possible in order to inform the design of trials in higher-level species or humans. A summary of all key input parameters for each study reviewed here is presented in **Table 1**.

**Table 1:** Key parameters in murine skin assessment studies related to a FLASH effect.

Study	Assay	Metric	Mouse model	Beam param	Avg Dose rate (Gy/s)	Total dose (Gy)	Fractionation	Anesthesia/gas	Location
Soto et al. (29)	Visual Skin toxicity at 8 weeks with histopathological post evaluations.	0 = normal 1 = <50% depigment. 2 = ≥50% depigment. 3 = <50% alopecia 4 = ≥50% alopecia 5 = <50% ulceration 6 = ≥50% ulceration	Female C57BL/6 mice	Electron, 16 MeV	CDR: 0.0747 UHDR: 180	10, 16, 20, 30, 40	1	Ketamine & xylazine.	Right hemithorax
Sorensen et al. (30)	Visual skin toxicity in 25 days Radiation-induced fibrosis (RIF) leg contraction model from 4-24 weeks.	1.5 = Moist desq. small/toes partly stuck togeth. >75% hair loss 2.0 = Moist desq. 25% Toes stuck together 2.5 = Moist desq. 50% Toes stuck & shape change. 3.0 = Moist desq. 75% Foot shapeless, 1 or 2 toes identified. 3.5 = Moist desq. entire area. Foot shapeless, no toes identified.	Female CDF1	Proton, 250 MeV, PBS	CDR: 0.33–0.66 UHDR: 81–89	40–60	1	None	Hind leg
Sorensen et al. (31)	Visual skin toxicity in 25 days	1.5 = Moist desq. small/toes partly stuck togeth. >75% hair loss 2.0 = Moist desq. 25% Toes stuck together 2.5 = Moist desq. 50% Toes stuck & shape change. 3.0 = Moist desq. 75% Foot shapeless, 1 or 2 toes identified. 3.5 = Moist desq. entire area. Foot shapeless, no toes identified.	Female CDF1	Proton, PBS, 250 MeV	CDR: 0.35–0.4 UHDR: 65–92	CDR: 23.2–39.2 FLASH: 31.2–53.5	1	None	Hind leg
Sorensen et al. (18)	Visual Skin toxicity in 25 days	1.5 = Moist desq. small/toes partly stuck togeth. >75% hair loss 2.0 = Moist desq. 25% Toes stuck together 2.5 = Moist desq. 50% Toes stuck & shape change. 3.0 = Moist desq. 75% Foot shapeless, 1 or 2 toes identified. 3.5 = Moist desq. entire area. Foot shapeless, no toes identified.	Female CDF1	Proton, PBS, 250 MeV	0.4 0.7 2 5.5 20 40 60 80	39.3	CDR: 1 × 39.3 6 × 6.6 UHDR: 1 × 39.3 2 × 19.7 3 × 13.1 4 × 9.8 6 × 6.6	None	Hind Leg
Duval et al. (32)	Visual Skin toxicity with histopathological post evaluations.	0: Normal pretreatment shaved mouse 1: Dry pre-moist desquamation 2/3: Partial/full thickness epidermal lysis/moist desquamation.	Female C57BL/6 mice	Electron, CDR: 9 MeV UHDR	CDR: 0.12 UHDR: 270	11–25	1 × 11 1 × 15 1 × 25 3 × 6 3 × 8	Isoflurane with 100% oxygen	Right flank
Rudigkeit et al. (33)	Ear thickness. Skin reaction via summation of desquamation and erythema scores for 180 days. Inflammation Cytokine analysis	Erythema Score: 0 = none 0.5 = Mild 1.5 = Definite 3.0 = severe Desquamation Score: 0 = None 1 = Dry 2 = Crust formation 3 = Moist	Female Balb/c mice	Proton, 20MeV	CDR: 0.06 lower UHDR: 9.3 UHDR: 930	23, 33	1	Medetomidine, midazolam, fentanyl.	Central part of the ear.
Mascia et al. (35)	Acute Skin toxicity via visual scoring Long term Skin toxicity via leg contracture.	1 = Normal 2 = alopecia 3 = erythema 4 = dry desquamation 5 = 30% moist desq. 6 = 70% moist desq.	Female C57Bl/6j	Proton, 250 MeV, PBS	CDR: 1 UHDR: 100	30, 35, 40, 45	1 × 30 1 × 35 1 × 40 1 × 45 2 × 15 2 × 17.5 3 × 10 3 × 11.6	Isoflurane	Right hindlimb
Zhang et al. (43)	Skin Contraction Histopathologic examination	Skin Contraction via the measurement between two ink dots. Histopathology was evaluated for epidermis thickness and collagen deposition.	Female FVB/N	Proton, 230 MeV	CDR: 0.4 UHDR: 130	25, 27, 30, 45	1	All mice: Ketamine/Xylzine. Set 1: Room air Set 2: Low Oxygen Set 3: Pure Oxygen.	Rear Leg.
Tavakkoli et al. (44)	Visual Skin toxicity.	# days to Ulceration.	Male and Female C57BL/6 mice	Electron, 9 MeV	CDR: 0.17 UHDR 200	27	1	Isoflurane Set 1: Room air Set 2: 100% Oxygen	Right Hind Leg
S. Cunningham et al.	• Acute plasma and skin TGF-β1 • Visual skin score • Delayed skin response via Cytokine Analysis • Leg contracture.	Visual Skin Scoring: 1 = Normal 2 = Alopecia 3 = Erythema 4 = Dry Desquamation 5 = <30% Moist Desq. 6 = >30% Moist Desq.	Female C57Bl/6j	Proton 250 MeV, PBS	CDR: 1 UHDR: 57 and 115	35	1	Isoflurane Room air	Right Hind leg

## 2 Review of studies and dosimetry effects

### 2.1 Dose rate effects

The dose rate is one of the key factors in FLASH skin-sparing efficacy. The first modern report of a dose–response study in murine skin toxicity was by Soto et al. (29), in 8 week old C57BL/6 mice anesthetized with ketamine/xylazine, who found that UHDR (180 Gy/s) irradiation led to both lower incidence and lower severity of skin ulceration than CDR (0.075 Gy/s). The response was assayed at 8 weeks after single-fraction hemi-thoracic irradiation, and differences were found at 30 Gy and 40 Gy, but not at doses of 10 Gy, 16 Gy, and 20 Gy. They suggested a shift in the dose–response curve for UHDR compared to CDR for this ulceration assay of the FLASH effect at single high-dose values of >20 Gy.

Sorensen et al. (18, 30, 31) conducted extensive studies on FLASH pencil beam scanning (PBS) 250 MeV proton irradiation in a 16–20-week-old CDF1 mouse model. Comparing CDR (0.33 Gy/s–0.63 Gy/s) and UHDR (71 Gy/s–89 Gy/s) to the murine foot with a total dose range of 23 Gy–60 Gy. They measured acute moist desquamation (MD), and one study (30) examined radiation-induced fibrosis as the measured endpoint, plotting data across a range of doses to show logistic function effects with dose. In a study quantifying both acute and long-term effects, both assays showed a sparing effect, with a dose modifying factor of >1.3, acute skin effects and 1.14 in long term fibrosis with doses ranging from 40 Gy to 60 Gy, depending on the study. Focusing on the acute damage data (31), a range of doses allowed them to quote a dose-modifying factor of 1.44–1.58, or approximately 50% more dose could be delivered by FLASH for equivalent biological damage. The dose values for observable acute skin damage were 24.7 Gy for CDR and 39.1 Gy for UHDR, for a dose modifying factor of 1.58. Most recently Sorensen et al. (18) examined varying dose rates (0.37 Gy/s–80 Gy/s) at a fixed 39.3 Gy total dose. The time structure of the PBS beam was also varied by introducing repainting of the field while keeping the field dose rate constant, which resulted in a higher dose rate needed to induce the FLASH effect. In this study, the dose rate was shown to have a sparing effect by the dose rate in which 50% (DR50) of the mice developing skin damage, wherein mild skin damage had a DR50 of 55 Gy/s, while severe skin damage showed a DR 50 of 2 Gy/s at an identical total dose. Thus, in this tissue model, the single-dose values for observation of skin sparing were quite high, but the conclusiveness of skin sparing was highly convincing at these levels.

Duval et al. (32), compared temporal kinetics and degree of flank skin damage and tumor response in 7–10-week-old C57BL/6 mice, using both single and fractionated dose delivery. Skin sparing was observed from a single dose of 25 Gy with electron beams UHDR (270 Gy/s) versus CDR (0.12 Gy/s), leading to a 7 day (21 ± 3days UHDR vs 29 ± 3 CDR, p-value = 0.02) increased latent period to MD, although no significant change was seen after 30 Gy dose. This latency of onset of skin damage points to a significant biological change rather than a simple dose-modifying effect.

Rudigkeit et al. (33) examined skin response in ear of Balb/c mice irradiated with 20 MeV protons CDR of 0.06 Gy/s and two UHDRs, 9.3 Gy/s and 930 Gy/s, using total dose of either 23 Gy or 33 Gy. Measurements of ear thickness, MD, and erythema were recorded, peaking approximately 3 weeks post-irradiation. This showed no difference in the 23 Gy group, but ear swelling and inflammation were reduced by 57% ± 12% and 67% ± 17% for the lower UHDR and 40% ± 13% and 50 ± 17% for the higher UHDR as compared to CDR.

Bohlen et al. (34) summarized data from many decades of FLASH studies to quantify the Dose-Modifying Factor of FLASH. They converted data from the CDR and UHDR effects to a common scale using isoeffect dose ratios and referred to them as the FLASH-modifying factor (FMF = (CDR/UHDR)|isoeffect). They found that FMF decreased with increased sparing as a function of the single-fraction dose. FMF values were 0.95 ± 0.11 for all data were <10 Gy, and 0.96 ± 0.07 (25 Gy) and 0.71 ± 0.06 (25 Gy). Thus, the magnitude of the effect is thought to be in the range of 29% for skin sparing, but only at higher single doses, based on the data reviewed.

Although not yet demonstrated in the skin, there is evidence of an incremental benefit in tissue-sparing with increasing dose rates above 30 Gy/s. Montay-Gruel et al. (17) irradiated whole brains of mice with 10 Gy of 6 MeV electrons at dose rates ranging from 0.1 Gy/s to 500 Gy/s. Cognitive function was assayed based on performance on a novel object recognition test. Sparing of cognitive performance was first observed at 30 Gy/s, with additional gains at 60 Gy/s and 100 Gy/s, a dose rate at which mice performed equally well as the non-irradiated controls. Although it is likely to be organ- and end-point-specific, this suggests that a threshold may exist after which increases in dose rate do not translate into clinically meaningful improvements in tissue sparing. More work using sensitive radiation damage assays across different organs is needed before definitive conclusions can be drawn.

### 2.2 Total dose per fraction and split dose studies

The doses used for observation of FLASH skin sparing have depended upon the mouse model, but also appear to vary by nearly a factor of 2 between different investigators. Further complications include the location of these skin assays (ear, hind leg, and tail), as well as the volume irradiated. The key factor is not necessarily that these specific dose levels are required for the benefit of FLASH, but perhaps that these doses are required to observe the benefit of FLASH in a particular skin response assay. However, this is the crux of the debate, if a large total single fraction dose is required to see the benefit of FLASH, or if this might also be observed with smaller fractions of dose, which would align with the common practice in clinical radiotherapy.

A critically important recent study by Mascia et al. (35) reported on proton UHDR in the skin, where reduced mouse skin toxicity and fibrosis were observed only for single, uninterrupted, high-dose fractions, and not for the same dose delivered in shorter split doses on the same day. This study utilized an approach of multiple split doses separated by 2 min each to examine how even short separations of time between split doses could reduce the skin-sparing effect. Irradiating the hind legs of C57Bl/6j mice at CDR (1 Gy/s) or UHDR (100 Gy/s) values, skin toxicity was scored skin at 7 weeks. Irradiation was either a single delivery or divided into two or three equal split doses with an interruption of 2 min. At a total dose of 35 Gy, splitting the dose in half (2 × 17.5 Gy) preserved the FLASH sparing effect, although this was not seen at 30 Gy (2 × 15 Gy). Choosing the splitting dose in three deliveries appeared to always negate the FLASH effect (3 × 10 Gy or 3 × 11.7 Gy), which is a seminal discovery that splitting doses are a new parameter that is shown to modulate the FLASH effect, and delivery of large total doses appears to be needed to see the benefits of FLASH. However, the choice of the number of splits and potentially conventional fractions is still convolved with the total dose delivered, and both are critical to see a difference in outcome between CDR and UHDR.

This was followed by Sorensen et al. (18) who studied split doses separated by an identical 2 min. The irradiation of hind legs of CDF1 mice to a total dose of 39.3 Gy at both CDR (0.37 Gy/s) and UHDR (60 Gy/s) with skin damage scored between 11 and 25 days. In this study doses were split in steps from 1 × 39.3 Gy, 2 × 19.7 Gy, 3 × 13.1 Gy, 4 × 9.8 Gy, and 6 × 6.6 Gy for UHDR showing an increase in sparing as the number of split doses decreases, while in CDR 1 × 39.3 Gy and 6 × 6.6 Gy showed no significant difference. Together, these studies help confirm that the total dose per split is a necessary planning parameter for inducing the FLASH effect.

The outcome of these experiments is not ideal for the value of translational FLASH because it suggests that fractionated delivery of UHDR can limit its efficacy in terms of skin sparing. This implies that the FLASH effect requires high total doses to be delivered in a short period of time, that is, faster than minutes. However, this study was carried out in mouse models with a high threshold for radiation damage and short-term metrics of damage. The timeline of split doses may be an extremely useful tool to determine the origins of FLASH, because it can be combined with the biological assay of skin sparing to determine the temporal kinetics of whatever causes the reduced damage. Further investigation of this is warranted in other models of radiation damage and clinical fractionation schemes.

## 3 Review of anesthesia and inspired gas effects

### 3.1 Oxygen and anesthesia

Oxygen is a well-known modulator of radiation damage (36, 37), and after the actual dose delivery, it is perhaps the largest effector of damage (38–40). The quantification of this has been conventionally described by the Oxygen Enhancement Ratio (OER) (39, 41), and this class has a value of 2.7 in *in vitro* cell death with full normal tissue oxygenation, as compared to air complete hypoxia. This effect has been observed *in vivo* in mouse skin (40); however, the OER value is highly variable with tissue type, and it should be noted that many tissues have temporally and spatially varying oxygen, which varies on a microscopic distance scale between capillaries. Thus, given all this complexity, making hard conclusions about the role of oxygen is challenging, especially for the skin, which has reasonably low regulation in the homeostasis of oxygen. The efficacy of FLASH sparing has been shown to be modulated and reduced by inhaled oxygen during anesthesia (42), and its effect on the skin has not been well documented until recently.

Zhang et al. (43) examined the proton FLASH effect (UHDR 130 Gy/s and CDR 0.4 Gy/s with 25 Gy and 30 Gy total dose) on skin shrinkage in FVB/N mice as marked by tattooed dots, and also by varying inhaled gas with 100% O_2_ versus air, and then ligated the leg to suppress blood flow effectively, achieving transient 0% oxygenation. UHDR irradiation resulted in a 15% reduction in skin contraction compared to CDR, with epidermal thickness and collagen deposition showing less damage to UHDR. Interestingly, both enhanced oxygen and restricted blood flow by ligation removed this dose-rate difference in the skin response. These data show the complexity of the role of oxygen in that high and zero values do not allow observation of the FLASH effect, but mid-level normoxia values do.

A critical observation in skin is that skin oxygen is highly variable and dependent upon the anesthesia and mouse physiology. Tavakkoli et al. (44) evaluated gender and gas anesthesia in the FLASH effect. C57BL/6 mice were anesthetized using isoflurane mixed with either room air or 100% oxygen. Mice that received 27 Gy of either UHDR or CDR and the time to ulceration were significantly shorter in mice that received 100% oxygen than in air, and female mice ulcerated sooner than males. The measured tissue oxygen was higher using 100% oxygen in the anesthesia carrier gas than in air, and female mice showed higher pO_2_ than males under 100% oxygen. Thus, UHDR skin-sparing required normal air to be used for breathing, suggesting that intermediate oxygen values were optimal for this effect (22–24).

### 3.2 Measuring *in vivo* tissue oxygenation

Other related measurements have shown that oxygen is depleted by radiation, and the level of this depletion has been quantified to be in the range of 0.1 mmHg/Gy–0.6 mmHg/Gy in FLASH radiotherapy (27, 45). These values also depend on the dose rate (25, 26) and, perhaps surprisingly, are a strong function of the initial oxygen present in the tissue. The large range of variation (0.1 mmHg/Gy–0.6 mmHg/Gy) is likely dominated by the latter issue, where tissue oxygenation, especially in skin, can be highly variable based upon the physiological condition of the animal. Fluctuations in the second to second timescales have been documented. The direct link between these observations or oxygen consumption and the FLASH effect *in vivo* remains unclear if there is a causative relationship, but there is some implication that they could be linked by oxygen consumption leading to reactive oxygen species that contribute to tissue damage and cell death (22–24). Conclusive work in this area remains to be done; however, given the low amount of oxygen consumption, the actual oxygenation drop from a FLASH dose of 20 Gy–30 Gy is in the range of 2 mmHg to 6 mmHg, which has shown both *in vivo* and *in vitro*. Thus, these values are not at the level of inducing radiobiological hypoxia in most normal tissues; therefore, the hypothesis that flash induces a lowering of the OER based upon the loss of ambient oxygen seems unlikely given these measured data. Additionally, if oxygen was lowered to induce a lower OER, it is likely to be more dominant in the tumor than in normally oxygenated tissues. The fact that an acute drop in oxygenation can be measured during UHDR irradiation and that this change is dose rate-dependent is an indicator that it may be a surrogate measure for the more complex radiation chemistry occurring *in vivo*.

## 4 Discussion

### 4.1 Dose rate, total dose, and oxygenation

The efficacy of FLASH skin sparing will become critical to understanding as translational human studies are underway at a number of research centers, because many will focus on studies of skin lesions as a safe choice for first in human work. The collective mouse data that exist has some important lessons of the dominant factors, although it comes with significant limitations as well, given how different mouse skin is to human skin. Much data appear to focus on average dose rates, and there is no clear conclusion on what dose rate is required for the FLASH effect, but most assume that higher dose rates are nearly always better than lower, but data suggest that there may not be benefits beyond dose rates above a certain level, although this remains to be studied in detail (17, 18). The dose rate is intertwined with the total dose delivered and the fractionation approach, but it is not clear if higher total doses are required to take advantage of the FLASH effects and/or if any fractionation scheme can be achieved, which preserves the efficacy as well. However, recent data by Mascia et al. suggest that two brief fractions or fields of delivery might be acceptable, although three fractions were apparently not as efficacious in sparing. Furthermore, Sorensen et al. (18) showed that fractionation reduces the FLASH effect, with some sparing still observed in six fields. Further study using this single approach is warranted, especially in larger animal translational studies.

The role of tissue oxygenation may also be critical, although making a conclusive discovery of its role has been challenging, largely because many measurements may not directly sample the right compartment of the tissue or the right time scale. Transient changes in oxygenation in the skin can occur on the second to second timescale; therefore, dealing with this as a factor is much more challenging than measuring stable dosimetric parameters of the irradiation. However, it is likely that oxygen has been a confounding factor in some FLASH studies, and a better understanding of both inspired gas and tissue oxygen is needed to clearly understand how UHDR effects are linked to the FLASH effect. Additionally, the transient depletion observed in oxygen *in vivo* during UHDR irradiation indicate that this measurement is possible. Second, it is plausibly linked to the magnitude of the FLASH effect. However, this hypothesis remains to be tested in mechanistic studies. However, given that oxygen is one of the few diagnostic biological measures that can be translated into humans in radiotherapy, it may be as critical as measurement of the delivered dose itself.

### 4.2 What is needed? Further testing of clinically-relevant dosages, late-effect outcomes, and large animal models

The data in murine skin are ideal scientific evidence to determine if there is a useful FLASH effect *in vivo*, but they do not clearly point to all the information needed for clinical translation. Measures of moist desquamation and initial ulceration are acute measures that are not fully representative of the concerns in human studies. Skin fibrosis is a late endpoint that can show features of damage that are more relevant to humans. Early measures that can be quantified, such as skin inflammation (33), erythema, and post-inflammatory hyperpigmentation, should be included (46, 47). Another key issue is that murine skin may not lead to a useful long-term assessment of skin toxicity events, where there can be a second phase of damage occurring months after the first repair phase. In mice, with an epidermis that is only three cell layers thick, less than half the thickness of the human epidermis, skin damage, and repair kinetics and characteristics likely differ in translationally important ways. Therefore, translation to higher-level organisms is essential, such as in porcine skin models. Porcine models are expensive, but are considered the standard for human translation to assess the types of long-term outcomes that are most concerning for human clinical trials and for testing the NCI Common Terminology Criteria for Adverse Events (CTCAE) skin toxicity scoring (28, 48). The ability to irradiate multiple areas on the same pig also allows for testing the efficacy of the FLASH effect while varying the discussed modulating factors and simultaneously reducing the between-animal variability inherent to murine studies. Several studies on pigs have been conducted and long-term data should be collected in the coming years.

Veterinary studies assaying skin damage in other larger animal models, such as feline and canine studies, are another valuable source of preclinical information on FLASH RT, predominantly because the skin is closer to human thickness and response. These studies are beneficial, as they can provide both tumor and skin tissue response data and have some inherent biological variability seen in human studies. However, these are limited because currently no large-scale CDR comparison to UHDR has been conducted in a veterinary large animal study. Most studies are phase I trials for clinical translation focusing primarily on safety as the endpoint, and none have addressed comparative efficacy (28, 48–51).

### 4.3 What are the opportunities? *In vivo* radiation chemistry-based dosimetry

FLASH UHDR irradiation presents an opportunity to directly measure radiation chemistry *in vivo* because the duration of irradiation is much faster than any biological phenomena. For example, the observation of an acute transient drop in oxygenation illustrates the occurrence of rapid radiation chemistry. The additional finding that this change is dependent on the dose rate and initial oxygenation is a fascinating part of quantifying what happens during radiotherapy. FLASH UHDR is a singular opportunity to utilize fast measurements *in vivo* to quantify what is happening in all radiotherapy and to hopefully parse out the mechanisms of what changes with dose rate and total dose in split dose choices. Translation of these measurements to humans is possible, providing molecular-specific information about radiotherapy. While oxygen is one parameter that can be measured all the way from solution work, through animals to humans, there may also be other parameters, such as acidity or free radical production.

## 5 Summary

The large amount of murine skin reaction data provides ideal scientific findings for translational work on UHDR FLASH radiotherapy. The data are promising, although what is missing is data on late toxicity events were assessed to determine the minimum fraction size needed to retain the benefit of normal skin sparing while allowing hypofractionated delivery of the radiation. The fractionation of these deliveries will greatly influence the biological outcome of late effects (20, 21), requiring an enhanced understanding of the link between acute and late effects, specifically if and how the FLASH effect translates to varying onsets. Translational work on pig skin and veterinary studies is needed to assess this, where long-term outcomes are assessed in fractionation testing studies. To date the threshold for the FLASH effect across dose levels in the skin seems to be reliable at UHDRs >40 Gy/s and total dose values >20 Gy with fraction sizes >10 Gy. Although this has been measured in mice, the assays need to be duplicated in skin models that mimic human skin more, and with longer-term outcome assays of skin damage. The observation that oxygen transients can be captured from the skin is a fascinating opportunity to directly probe the radiation chemistry changes that occur during UHDR irradiation, and further translational measurements in humans are feasible. Together, these mechanistic studies of oxygen consumption with skin reddening or damage might help provide quantitative biomarkers of the FLASH effect that have direct relevance to human dosimetry.

## References

[1] Favaudon V Caplier L Monceau V Pouzoulet F Sayarath M Fouillade C. Ultrahigh dose-rate FLASH irradiation increases the differential response between normal and tumor tissue in mice. Sci Transl Med. (2014) 6:245ra93. doi: 10.1126/scitranslmed.3008973 25031268

[2] Montay-Gruel P Acharya MM Petersson K Alikhani L Yakkala C Allen BD. Long-term neurocognitive benefits of FLASH radiotherapy driven by reduced reactive oxygen species. Proc Natl Acad Sci U.S.A. (2019) 116:10943–51. 10.1073/pnas.1901777116 PMC6561167 31097580

[3] Limoli CL Vozenin MC. Reinventing radiobiology in the light of FLASH radiotherapy. Ann Rev Cancer Biol. (2023) 7:1–21. doi: 10.1146/annurev-cancerbio-061421-022217 39421564 PMC11486513

[4] Chabi S To THV Leavitt R Poglio S Jorge PG Jaccard M. Ultra-high-dose-rate FLASH and conventional-dose-rate irradiation differentially affect human acute lymphoblastic leukemia and normal hematopoiesis. Int J Radiat Oncol Biol Phys. (2021) 109:819–29. doi: 10.1016/j.ijrobp.2020.10.012 33075474

[5] Bourhis J Montay-Gruel P Jorge Goncalves P Bailat C Petit B Ollivier J. Clinical translation of FLASH radiotherapy: Why and how? Radiother Oncol. (2019) 139:11–7. 10.1016/j.radonc.2019.04.008 31253466

[6] Buchsbaum JC Coleman CN Espey MG Prasanna PGS Capala J Ahmed MM. FLASH radiation therapy: new technology plus biology required. Int J Radiat Oncol Biol Phys. (2021) 110:1248–9. doi: 10.1016/j.ijrobp.2021.01.053 33548337

[7] Kinj R Gaide O Jeanneret-Sozzi W Dafni U Viguet-Carrin S Sagittario E. Randomized phase II selection trial of FLASH and conventional radiotherapy for patients with localized cutaneous squamous cell carcinoma or basal cell carcinoma: A study protocol. Clin Transl Radiat Oncol. (2024) 45:100743. doi: 10.1016/j.ctro.2024.100743 38362466 PMC10867306

[8] Daugherty EC Zhang Y Xiao Z Mascia AE Sertorio M Woo J. FLASH radiotherapy for the treatment of symptomatic bone metastases in the thorax (FAST-02): protocol for a prospective study of a novel radiotherapy approach. Radiat Oncol. (2024) 19:34. doi: 10.1186/s13014-024-02419-4 38475815 PMC10935811

[9] Bourhis J Sozzi WJ Jorge PG Gaide O Bailat C Duclos F. Treatment of a first patient with FLASH-radiotherapy. Radiother Oncol. (2019) 139:18–22. doi: 10.1016/j.radonc.2019.06.019 31303340

[10] Daugherty EC Mascia A Zhang Y Lee E Xiao Z Sertorio M. FLASH radiotherapy for the treatment of symptomatic bone metastases (FAST-01): protocol for the first prospective feasibility study. JMIR Res Protoc. (2023) 12:e41812. doi: 10.2196/41812 36206189 PMC9893728

[11] Lourenco A Subiel A Lee N Flynn S Cotterill J Shipley D. Absolute dosimetry for FLASH proton pencil beam scanning radiotherapy. Sci Rep. (2023) 13:2054. doi: 10.1038/s41598-023-28192-0 36739297 PMC9899251

[12] Mascia AE Daugherty EC Breneman JC. FLASH radiotherapy in a value-based health care environment-reply. JAMA Oncol. (2023) 9:727. doi: 10.1001/jamaoncol.2023.0134 36951830

[13] Vozenin MC Bourhis J Durante M. Towards clinical translation of FLASH radiotherapy. Nat Rev Clin Oncol. (2022) 19:791–803. doi: 10.1038/s41571-022-00697-z 36303024

[14] Cengel KA Kim MM Diffenderfer ES Busch TM. FLASH radiotherapy: what can FLASH’s ultra high dose rate offer to the treatment of patients with sarcoma? Semin Radiat Oncol. (2024) 34:218–28. 10.1016/j.semradonc.2024.02.001 PMC12778364 38508786

[15] Diffenderfer ES Verginadis II Kim MM Shoniyozov K Velalopoulou A Goia D. Design, implementation, and in vivo validation of a novel proton FLASH radiation therapy system. Int J Radiat Oncol Biol Phys. (2020) 106:440–8. doi: 10.1016/j.ijrobp.2019.10.049 PMC7325740 31928642

[16] Pottern CS. Radiation and the Skin Vol. 237. London: Talyor & Francis Ltd (1985), 237.

[17] Montay-Gruel P Petersson K Jaccard M Boivin G Germond JF Petit B. Irradiation in a flash: Unique sparing of memory in mice after whole brain irradiation with dose rates above 100Gy/s. Radiother Oncol. (2017) 124:365–9. doi: 10.1016/j.radonc.2017.05.003 28545957

[18] Sorensen BS Kanouta E Ankjaergaard C Kristensen L Johansen JG Sitarz MK. Proton FLASH: impact of dose rate and split dose on acute skin toxicity in a murine model. Int J Radiat Oncol Biol Phys. (2024) 120:265–75. doi: 10.1016/j.ijrobp.2024.04.071 38750904

[19] Vozenin MC Hendry JH Limoli CL. Biological benefits of ultra-high dose rate FLASH radiotherapy: sleeping beauty awoken. Clin Oncol (R Coll Radiol). (2019) 31:407–15. doi: 10.1016/j.clon.2019.04.001 PMC6850216 31010708

[20] Bohlen TT Germond JF Bourhis J Bailat C Bochud F Moeckli R. The minimal FLASH sparing effect needed to compensate the increase of radiobiological damage due to hypofractionation for late-reacting tissues. Med Phys. (2022) 49:7672–82. 10.1002/mp.15911 PMC10087769 35933554

[21] Majeed H Gupta V. Adverse effects of radiation therapy. Treasure Island (FL): StatPearls (2024). 33085406

[22] Adrian G Konradsson E Lempart M Back S Ceberg C Petersson K. The FLASH effect depends on oxygen concentration. Br J Radiol. (2020) 93:20190702. doi: 10.1259/bjr.20190702 31825653 PMC7055454

[23] Petersson K Adrian G Butterworth K McMahon SJ. A quantitative analysis of the role of oxygen tension in FLASH radiation therapy. Int J Radiat Oncol Biol Phys. (2020) 107:539–47. doi: 10.1016/j.ijrobp.2020.02.634 32145319

[24] Poulsen PR Johansen JG Sitarz MK Kanouta E Kristensen L Grau C. Oxygen enhancement ratio-weighted dose quantitatively describes acute skin toxicity variations in mice after pencil beam scanning proton FLASH irradiation with changing doses and time structures. Int J Radiat Oncol Biol Phys. (2024) 120:276–86. doi: 10.1016/j.ijrobp.2024.02.050 38462015

[25] Van Slyke AL El Khatib M Velalopoulou A Diffenderfer E Shoniyozov K Kim MM. Oxygen monitoring in model solutions and *in vivo* in mice during proton irradiation at conventional and FLASH dose rates. Radiat Res. (2022) 198:181–9. 10.1667/RADE-21-00232.1 PMC10176203 35640166

[26] El Khatib M Van Slyke AL Velalopoulou A Kim MM Shoniyozov K Allu SR. Ultrafast tracking of oxygen dynamics during proton FLASH. Int J Radiat Oncol Biol Phys. (2022) 113:624–34. doi: 10.1016/j.ijrobp.2022.03.016 PMC9250619 35314293

[27] Cao X Zhang R Esipova TV Allu SR Ashraf R Rahman M. Quantification of oxygen depletion during FLASH irradiation in vitro and *in vivo* . Int J Radiat Oncol Biol Phys. (2021) 111:240–8. doi: 10.1016/j.ijrobp.2021.03.056 PMC8338745 33845146

[28] Vozenin MC De Fornel P Petersson K Favaudon V Jaccard M Germond JF. The advantage of FLASH radiotherapy confirmed in mini-pig and cat-cancer patients. Clin Cancer Res. (2019) 25:35–42. doi: 10.1158/1078-0432.CCR-17-3375 29875213

[29] Soto LA Casey KM Wang J Blaney A Manjappa R Breitkreutz D. FLASH irradiation results in reduced severe skin toxicity compared to conventional-dose-rate irradiation. Radiat Res. (2020) 194:618–24. doi: 10.1667/RADE-20-00090 PMC7855987 32853385

[30] Sorensen BS Sitarz MK Ankjaergaard C Johansen JG Andersen CE Kanouta E. Pencil beam scanning proton FLASH maintains tumor control while normal tissue damage is reduced in a mouse model. Radiother Oncol. (2022) 175:178–84. doi: 10.1016/j.radonc.2022.05.014 35595175

[31] Singers Sorensen B Krzysztof Sitarz M Ankjaergaard C Johansen J Andersen CE Kanouta E. In vivo validation and tissue sparing factor for acute damage of pencil beam scanning proton FLASH. Radiother Oncol. (2022) 167:109–15. doi: 10.1016/j.radonc.2021.12.022 34953933

[32] Duval KEA Aulwes E Zhang R Rahman M Ashraf MR Sloop A. Comparison of tumor control and skin damage in a mouse model after ultra-high dose rate irradiation and conventional irradiation. Radiat Res. (2023) 200:223–31. doi: 10.1667/RADE-23-00057 PMC10551764 37590482

[33] Rudigkeit S Schmid TE Dombrowsky AC Stolz J Bartzsch S Chen CB. Proton-FLASH: effects of ultra-high dose rate irradiation on an *in-vivo* mouse ear model. Sci Rep. (2024) 14:1418. doi: 10.1038/s41598-024-51951-6 38228747 PMC10791610

[34] Bohlen TT Germond JF Bourhis J Vozenin MC Ozsahin EM Bochud F. Normal tissue sparing by FLASH as a function of single-fraction dose: A quantitative analysis. Int J Radiat Oncol Biol Phys. (2022) 114:1032–44. doi: 10.1016/j.ijrobp.2022.05.038 35810988

[35] Mascia A McCauley S Speth J Nunez SA Boivin G Vilalta M. Impact of multiple beams on the FLASH effect in soft tissue and skin in mice. Int J Radiat Oncol Biol Phys. (2024) 118:253–61. doi: 10.1016/j.ijrobp.2023.07.024 37541394

[36] Van den Brenk HA Kerr RC Richter W Papworth MP. Enhancement of radiosensitivity of skin of patients by high pressure oxygen. Br J Radiol. (1965) 38:857–64. doi: 10.1259/0007-1285-38-455-857 5842583

[37] Molls M Stadler P Becker A Feldmann HJ Dunst J. Relevance of oxygen in radiation oncology. Mechanisms of action, correlation to low hemoglobin levels. Strahlenther Onkol. (1998) 174(Suppl 4):13–6. 9879341

[38] Okunieff P Hoeckel M Dunphy EP Schlenger K Knoop C Vaupel P. Oxygen tension distributions are sufficient to explain the local response of human breast tumors treated with radiation alone. Int J Radiat Oncol Biol Phys. (1993) 26:631–6. doi: 10.1016/0360-3016(93)90280-9 8330993

[39] Beck-Bornholdt HP Dubben HH. Oxygen enhancement ratio of a murine fibrosarcoma. Int J Radiat Oncol Biol Phys. (1992) 24:805–6. doi: 10.1016/0360-3016(92)90735-Z 1429111

[40] Hendry JH Moore JV Hodgson BW Keene JP. The constant low oxygen concentration in all the target cells for mouse tail radionecrosis. Radiat Res. (1982) 92:172–81. doi: 10.2307/3575852 7134382

[41] Skarsgard LD Harrison I. Dose dependence of the oxygen enhancement ratio (OER) in radiation inactivation of Chinese hamster V79-171 cells. Radiat Res. (1991) 127:243–7. doi: 10.2307/3577937 1886978

[42] Iturri L Bertho A Lamirault C Brisebard E Juchaux M Gilbert C. Oxygen supplementation in anesthesia can block FLASH effect and anti-tumor immunity in conventional proton therapy. Commun Med (Lond). (2023) 3:183. doi: 10.1038/s43856-023-00411-9 38102219 PMC10724215

[43] Zhang Q Gerweck LE Cascio E Yang Q Huang P Niemierko A. Proton FLASH effects on mouse skin at different oxygen tensions. Phys Med Biol. (2023) 68. doi: 10.1088/1361-6560/acb888 PMC11164666 36731139

[44] Tavakkoli AD Clark MA Kheirollah A Sloop AM Soderholm HE Daniel NJ. Anesthetic oxygen use and sex are critical factors in the FLASH sparing effect. bioRxiv. (2023). doi: 10.1101/2023.11.04.565626 PMC11070800 38711960

[45] Petusseau AF Clark M Bruza P Gladstone D Pogue BW. Intracellular oxygen transient quantification in vivo during ultra-high dose rate FLASH radiation therapy. Int J Radiat Oncol Biol Phys. (2024). doi: 10.1016/j.ijrobp.2024.04.068 PMC12012821 38703954

[46] Matsubara H Matsufuji N Tsuji H Yamamoto N Karasawa K Nakajima M. Objective assessment in digital images of skin erythema caused by radiotherapy. Med Phys. (2015) 42:5568–77. doi: 10.1118/1.4928890 26329003

[47] Nystrom J Svensk AC Lindholm-Sethson B Geladi P Larson J Franzen L. Comparison of three instrumental methods for the objective evaluation of radiotherapy induced erythema in breast cancer patients and a study of the effect of skin lotions. Acta Oncol. (2007) 46:893–9. 10.1080/02841860701243087 17917821

[48] Rohrer Bley C Wolf F Jorge Goncalves P Grilj V Petridis I Petit B. Dose- and volume-limiting late toxicity of FLASH radiotherapy in cats with squamous cell carcinoma of the nasal planum and in mini pigs. Clin Cancer Res. (2022) 28:3814–23. doi: 10.1158/1078-0432.CCR-22-0262 PMC9433962 35421221

[49] Gjaldbaek BW Arendt ML Konradsson E Jensen Bastholm K Back SAJ Munck Af Rosenschold P. Long-term toxicity and efficacy of FLASH radiotherapy in dogs with superficial Malignant tumors. Front Oncol. (2024) 14:1425240. doi: 10.3389/fonc.2024.1425240 39077466 PMC11284943

[50] Konradsson E Arendt ML Bastholm Jensen K Borresen B Hansen AE Back S. Establishment and initial experience of clinical FLASH radiotherapy in canine cancer patients. Front Oncol. (2021) 11:658004. doi: 10.3389/fonc.2021.658004 34055624 PMC8155542

[51] Velalopoulou A Karagounis I Cramer GM Kim MM Skoufos G Goia D. FLASH proton radiotherapy spares normal epithelial and mesenchymal tissues while preserving sarcoma response. Cancer Res. (2021) 81:4808–21. doi: 10.1158/0008-5472.CAN-21-1500 PMC8715480 34321243

